# *Wickerhamomyces anomalous*: A Rare Cause of Fungemia Causing Febrile Neutropenia in Acute Lymphoblastic Leukemia

**DOI:** 10.1155/2020/8847853

**Published:** 2020-12-29

**Authors:** Vibha Mehta, Aroop Mohanty, Suneeta Meena, J. S. Rahul, Nath Uttam Kumar, Debranjani Chattopadhyay, Anamika Bakliwal, Ranjana Choudhary, Pratima Gupta

**Affiliations:** ^1^PDCC Hepatic and Transplant Virology, ILBS, New Delhi, India; ^2^Department of Clinical Microbiology, AIIMS, Gorakhpur, India; ^3^Department of Laboratory Medicine, AIIMS, New Delhi, India; ^4^Department of Neuroanaesthesia and Critical Care, AIIMS, New Delhi, India; ^5^Department of Medical Oncology and Haematology, AIIMS Rishikesh, Rishikesh, India; ^6^Department of Microbiology, AIIMS Rishikesh, Rishikesh, India

## Abstract

*Candida* bloodstream infection is the major cause of increased morbidity and mortality (20–49%) in hospitalized patients in both paediatric and adult age groups. Due to the increase in the number of immunocompromised patients, other important species such as *Trichosporon asahii* and *Debaryomyces hansenii* are emerging. One such organism, *Wickerhamomyces anomalous*, previously known as *Pichia anomala* (teleomorph stages of several *Candida* species), is increasingly being reported as a cause of fungemia in neonatal intensive care units and is now increasingly being reported in a lot of immunosuppressive conditions such as interstitial lung disease, endocarditis, enteritis, corticosteroids, and chemotherapy uptake. Though this yeast is ubiquitous in nature, systemic infections from isolated cases and sporadic outbreaks with high mortality have been reported in ICUs, which emphasize the importance to consider this fungus within the diagnostic possibilities. Here, we report a case of catheter-related bloodstream infection (CRBSI) caused by *W. anomalus* in a leukemic immunosuppressed patient who was successfully treated by early detection and treatment of this emerging fungus.

## 1. Introduction

*Candida* bloodstream infection is the primary cause of increased morbidity and mortality (20–49%) in hospitalized patients in both paediatric and adult age groups worldwide. Despite advancements in the early diagnosis and treatment, *Candida* species rank fourth in the United States and seventh in Europe in causing bloodstream infections (BSIs) [1]. In India, few studies have reported varying candidemia rates from 4 to as high as 18% with a constant increase in isolation of non-albicans *Candida* (NAC) from BSIs [1]. Initially, only *Candida albicans* were considered to be the most crucial yeast species to cause invasive disseminated infection; however, due to an increase in the number of immunocompromised patients, other important yeast species such as *Trichosporon asahii* and *Debaryomyces hansenii* are emerging rampantly [2].

*Wickerhamomyces anomalus* (*W. anomalus*), previously known as *Hansenula anomala* (*H. anomala*), was later on included as a synonym in the genus *Pichia* and was referred to as *Pichia anomala* [3, 4]. It is the teleomorph stage of several *Candida* species and is found mainly in plants, fruits, and oil. The first report about *H. anomala* isolation from the lungs of an infant was published in 1958 by Wang and Schwarz [5]. In India, it was first responsible for causing an outbreak in the neonatal intensive care unit of a tertiary care centre in 1996. It is now being increasingly reported in a lot of immunosuppressive conditions such as interstitial lung disease, endocarditis, enteritis, corticosteroids, and chemotherapy uptake [6]. Though this yeast is ubiquitous in nature, systemic infections from isolated cases and sporadic outbreaks with high mortality have been reported in ICUs, which emphasize the importance to consider this fungus within the diagnostic possibilities [6]. Here, we report a case of catheter-related bloodstream infection (CRBSI) caused by *W. anomalus* in a leukemic immunosuppressed patient who was successfully treated by early detection and treatment of this emerging fungus.

## 2. Case Report

**Figure 1:**
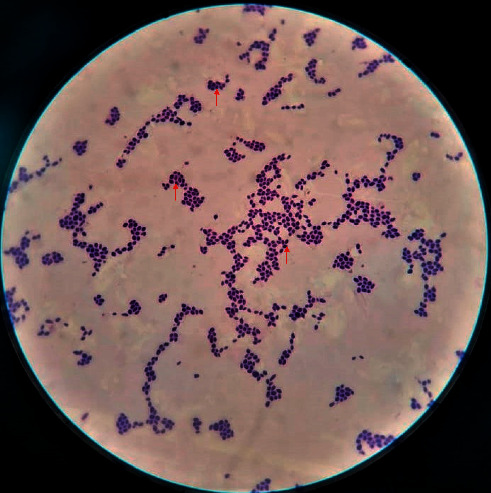
Direct Gram stain from both positive blood culture bottles revealed budding yeast cells (BYCs).

**Figure 2:**
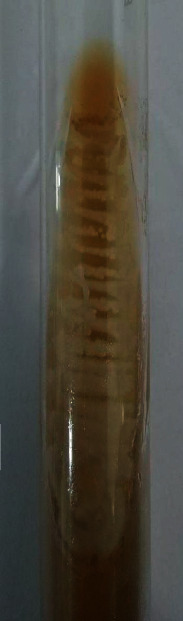
White to cream-colored, glabrous yeast-like colonies of *W. anomalus* were formed on SDA at 37°C after 24 hours of incubation.

**Figure 3:**
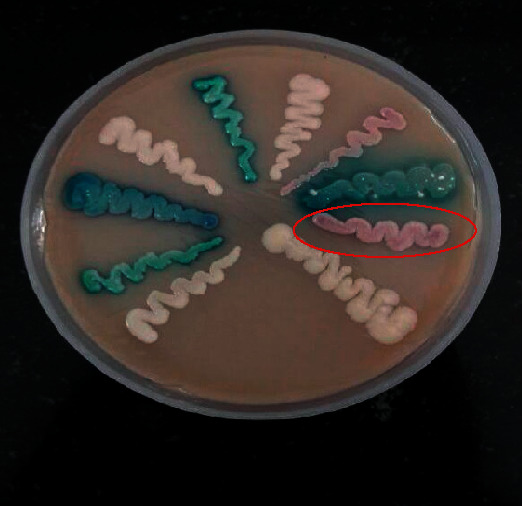
CHROM agar Candida showed the presence of pink mucoid colonies of *W. anomalus* grown after 48 hours at 37°C.

A 36-year-old male, follow-up case of B-cell acute lymphoblastic leukemia on cyclophosphamide and methotrexate immunosuppressive therapy, presented to the emergency department of our tertiary care centre with the chief complaint of fever with chills and rigors for two days. The fever started four days after the last chemotherapy session. The patient had an implanted long-term central venous catheter. Previous chemotherapy sessions were uneventful. Radiographic and laboratory evaluations (including CBC, LFT, and RFT) were unremarkable. Blood culture from both central catheter and peripheral line was collected and sent to the microbiology laboratory. He was treated empirically with injective piperacillin-tazobactam 3.375 gm IV 6 hourly and injection vancomycin 1 gm IV 12 hourly. After 22 hours of incubation, the BACTEC blood culture system (Becton Dickinson and Company, USA) signalled positive in both central catheter and peripheral line. However, the central line came out to be positive one hour before the peripheral line. Direct Gram stain from both positive blood culture bottles revealed budding yeast cells (Figure 1). The specimen was subcultured onto blood agar, chocolate agar, MacConkey agar, and Sabouraud's dextrose agar (SDA) at 27°C and 34°C, respectively. Within 24 hours, white to cream-colored, glabrous yeast-like colonies of *W. anomalus* were formed on SDA at 37°C (Figure 2). Conventional methods such as lactophenol cotton blue (LPCB) mount and cornmeal agar (CMA) showed spherical to ellipsoidal budding yeast cells with abundant pseudohyphae. The germ tube test came out to be negative. CHROMagar Candida showed the presence of pink mucoid colonies of *W. anomalus* grown after 48 hours at 37°C (Figure 3). It was further identified by MALDI-TOF MS (Bruker Daltonics, Bremen, Germany) as *Wickerhamomyces anomalus* with a confidence interval (CI) of 2.19. Hence, a clinical diagnosis of *W. anomalus* catheter-related bloodstream infection (CRBSI) was established. Fluconazole (800 mg on the first day followed by 400 mg O.D.) was started, and antibiotics were de-escalated. The central venous catheter and peripheral line were removed immediately, and the tip culture was sent for fungal culture, which also yielded positive growth for *W. anomalus*. No other focus of *Wickerhamomyces* could be noticed. After three days of antifungal therapy, the patient clinically improved and became afebrile. A follow-up blood culture after two weeks was performed, which came out to be sterile, and as a result, the patient was discharged under satisfactory conditions.

## 3. Discussion

*Candida albicans* has always been in the limelight as essential yeast species to cause human invasive infections. However, due to increased immunosuppressive conditions and advanced methods for detection and identification of yeasts, many other clinically critical opportunistic species are reported with varying antifungal susceptibility [7].

*W. anomalus* is a ubiquitous organism and was initially thought to be a plant pathogen, but recently, it has been increasingly reported as a causative agent of fungemia in both immunocompetent and immunocompromised patients [8, 9]. It has been reported as a causative agent of nosocomial fungemia in neonatal intensive care units, critical care units, and AIDS patients. It has also been implicated in nosocomial cerebral ventriculitis in low-birth-weight neonates, endocarditis in intravenous drug abusers, and urinary tract infection in renal transplant recipients. Cross-contamination, through the hands of caregivers, has also been incriminated as an important cause in the past [10–13]. In our case, the presence of an implanted central venous catheter in the adult patient on chemotherapy could be a possible contributory risk factor for the fungus to grow.

The literature on the susceptibility profile of *W. anomalus* is limited; though based on the susceptibility profiles of most isolates, it appears to be very much similar to that of *Candida glabrata* [14]. Several studies were carried out to analyse the antifungal susceptibility of clinical isolates belonging to this uncommon species of *Candida*. One study showed that out of fifteen *W*. *anomalus* strains, eight were resistant to fluconazole, six to itraconazole, and ketoconazole to both, and one was flucytosine resistant [15].

Da Matta et al. were able to publish the most extensive susceptibility profile of such isolates from patients presenting with nosocomial fungemia where they reported MICs of 16, 1, 0.5, 0.25, and 1 to fluconazole, itraconazole, voriconazole, caspofungin, and amphotericin B, respectively. A series of case reports by Chitasombat et al. describes one isolate of *W. anomalus* that was susceptible to the azoles, amphotericin B, and echinocandins [16].

These findings are quite worrisome since besides amphotericin B, only a few therapeutic options are currently effective against this rare fungal infection. Although antifungal susceptibility testing could not be performed in our case, our patient was treated successfully with fluconazole therapy for 14 days.

This case further puts a much-needed light on *W. anomalus* and its ability to cause invasive infection with varying antifungal susceptibility. It is rather challenging to identify *W. anomalus* by conventional as well as widely used commercial yeast diagnostic kits such as API 20C AUX, ID32C, and Vitek-2 and commonly misidentified as *Candida utilis* by commercial systems [17].

In our patient, the pathogen was identified as *W. anomalus* by MALDI-TOF MS (Bruker Daltonics, Bremen, Germany) which yielded the results in a few minutes and helped the clinicians in early initiation of the therapy of choice and improving the prognosis in this case. Molecular identification was not possible in this case due to limited resources and facilities. Our experience with these rare yeasts is that antifungal susceptibility-driven therapy is the key to management of such pathogens.

Our case report demonstrates the importance of early suspicion, organism identification, and differentiation of ascomycetous yeast species, specifically in immunosuppressed high-risk patient populations such as those with total parenteral nutrition, term and preterm NICU patients, chronic indwelling intravenous access, and compromised immune systems. An effective antifungal prophylaxis policy may be necessary for these patients when one or more risk factors exist.

## 4. Conclusion

With an emerging number of fungal pathogens in these patient populations that are not reliably sensitive to azoles, it is increasingly necessary to cultivate a healthy suspicion for echinocandins and azole-resistant organisms that may further require susceptibility testing. So, early suspicion, detection, and treatment can be very helpful in curbing these emerging pathogens.
